# Targeting Nrf2 to Suppress Ferroptosis and Mitochondrial Dysfunction in Neurodegeneration

**DOI:** 10.3389/fnins.2018.00466

**Published:** 2018-07-10

**Authors:** Moataz Abdalkader, Riikka Lampinen, Katja M. Kanninen, Tarja M. Malm, Jeffrey R. Liddell

**Affiliations:** ^1^A.I. Virtanen Institute for Molecular Sciences, University of Eastern Finland, Kuopio, Finland; ^2^Department of Pharmacology and Therapeutics, The University of Melbourne, Parkville, VIC, Australia

**Keywords:** Alzheimer’s disease, Parkinson’s disease, Huntington’s disease, motor neuron disease, RSL3, erastin, Keap1, system x_c_^-^

## Abstract

Ferroptosis is a newly described form of regulated cell death, distinct from apoptosis, necroptosis and other forms of cell death. Ferroptosis is induced by disruption of glutathione synthesis or inhibition of glutathione peroxidase 4, exacerbated by iron, and prevented by radical scavengers such as ferrostatin-1, liproxstatin-1, and endogenous vitamin E. Ferroptosis terminates with mitochondrial dysfunction and toxic lipid peroxidation. Although conclusive identification of ferroptosis *in vivo* is challenging, several salient and very well established features of neurodegenerative diseases are consistent with ferroptosis, including lipid peroxidation, mitochondrial disruption and iron dysregulation. Accordingly, interest in the role of ferroptosis in neurodegeneration is escalating and specific evidence is rapidly emerging. One aspect that has thus far received little attention is the antioxidant transcription factor nuclear factor erythroid 2-related factor 2 (Nrf2). This transcription factor regulates hundreds of genes, of which many are either directly or indirectly involved in modulating ferroptosis, including metabolism of glutathione, iron and lipids, and mitochondrial function. This potentially positions Nrf2 as a key deterministic component modulating the onset and outcomes of ferroptotic stress. The minimal direct evidence currently available is consistent with this and indicates that Nrf2 may be critical for protection against ferroptosis. In contrast, abundant evidence demonstrates that enhancing Nrf2 signaling is potently neuroprotective in models of neurodegeneration, although the exact mechanism by which this is achieved is unclear. Further studies are required to determine to extent to which the neuroprotective effects of Nrf2 activation involve the prevention of ferroptosis.

## Ferroptosis; an Iron-Dependent Non-Apoptotic Form of Regulated Cell Death

The last few decades have witnessed a surge in the discovery of new forms of regulated cell death that have immense implications for both health and disease (Galluzzi et al., 2018). Ferroptosis is a recently described form of non-apoptotic regulated cell death caused by uncontrolled iron-dependent lipid peroxidation that is distinct in its morphological, biochemical, and genetic profile from other cell death mechanisms (Dixon et al., 2012; Galluzzi et al., 2018). Cells undergoing ferroptosis show none of the classical morphological alterations associated with apoptosis, necroptosis, or autophagy (e.g., cell swelling, nuclear disruption, membrane blebbing, etc.), with the only discernable ultrastructural feature being distinctly altered mitochondrial morphology (Dixon et al., 2012; Stockwell et al., 2017).

**FIGURE 1:**
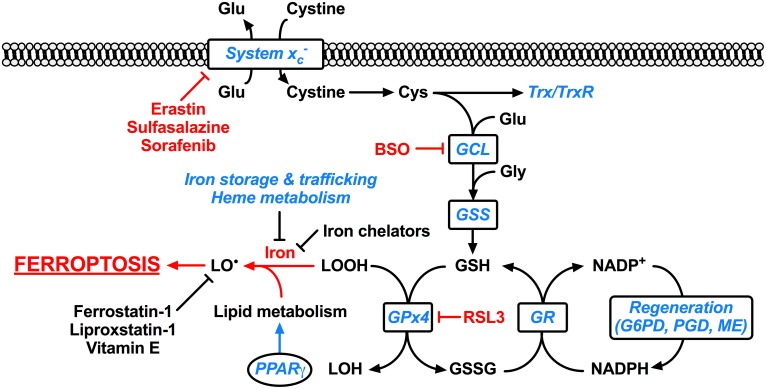
Ferroptosis and its molecular regulation by Nrf2. Glutathione peroxidase 4 (Gpx4) utilizes the major cellular antioxidant glutathione (GSH) as a substrate to reduce lipid hydroperoxides (LOOH). Oxidized glutathione (GSSG) generated by Gpx4 is reduced back to glutathione by glutathione reductase (GR) in a reaction requiring NADPH, which can be regenerated by glucose-6-phosphate dehydrogenase (G6PD) and phosphogluconate dehydrogenase (PGD) of the pentose-phosphate pathway, and malic enzyme (ME). The tripeptide glutathione is synthesized by the consecutive action of glutamate-cysteine ligase (GCL) and glutathione synthetase (GSS), where the ligation of cysteine (Cys) and glutamate (Glu) by glutamate-cysteine ligase is the rate-limiting step of glutathione synthesis. The cellular import of cystine by the glutamate-cystine antiporter system x_c_^-^ constitutes an significant route of cysteine supply for glutathione synthesis. Cysteine is also required for other important cellular antioxidants including thioredoxin (Trx) and thioredoxin reductase (TrxR). Ferroptosis occurs when the Gpx4-catalyzed reduction of lipid hydroperoxides is insufficient to prevent the iron-mediated generation of lipid radicals (LO^∙^). This leads to the propagation of lipid peroxidation and culminates in ferroptosis. Ferroptosis can be experimentally induced by inhibiting Gpx4 via the small molecule inhibitor RSL3, or by limiting glutathione supply to Gpx4. The latter is induced by direct [e.g., buthionine sulfoximine (BSO)] or indirect (e.g., by limiting cysteine availability) inhibition of glutathione synthesis. Cysteine supply is disrupted by inhibitors of system x_c_^-^ including erastin, sulfasalazine, and sorafenib. Ferroptosis can also be inhibited by iron chelators such as deferoxamine and deferiprone, and lipid radical scavengers such as ferrostatin-1, liproxstatin-1, and vitamin E. Factors transcriptionally regulated by Nrf2 are indicated in blue italics, whereas factors promoting ferroptosis are indicated in red. Clearly Nrf2 signaling is likely to have an integral and pervasive impact on the manifestation of ferroptosis.

A discriminating feature of ferroptosis is the potent capacity of lipid peroxide scavengers (such as ferrostatin-1, liproxstatin-1, and vitamin E) to prevent ferroptotic cell death (Dixon et al., 2012; Friedmann Angeli et al., 2014; Yang et al., 2014). This explicit requirement for lipid peroxidation is a core feature of ferroptosis that distinguishes it from other forms of cell death: ferroptosis inhibitors cannot prevent other forms of cell death (Dixon et al., 2012; Friedmann Angeli et al., 2014; Yang et al., 2014), and conversely classic inhibitors of necrosis, apoptosis, and autophagy do not modulate ferroptosis (Dixon et al., 2012), with the exception of the necroptosis inhibitor, necrostatin-1 (RIPK1 inhibitor), which can inhibit ferroptosis in a necroptosis/RIPK1-independent manner (Friedmann Angeli et al., 2014). The central endogenous suppressor of ferroptosis is the selenoenzyme glutathione peroxidase 4 (Gpx4). Gpx4 detoxifies membrane lipid hydroperoxides, preventing unchecked toxic lipid peroxidation. Gpx4 requires the major cellular antioxidant glutathione as a substrate, and hence ferroptosis is also dependent on glutathione levels (Friedmann Angeli et al., 2014). The precise role of iron in ferroptosis is ironically unclear (see below), however, its involvement is unequivocally indicated by the strong inhibition of cell death associated with iron chelation or limiting iron availability (**Figure 1**).

Ferroptosis appears to require the presence of specific highly oxidisable phosphatidylethanolamine phospholipids containing the polyunsaturated fatty acids (PUFAs) arachidonic acid and adrenic acid. Acyl-CoA synthetase long-chain family member 4 (ACSL4) is important for the synthesis of phospholipids from these PUFAs, while lysophosphatidylcholine acyltransferase 3 (LPCAT3) is important for their insertion into membrane phospholipids (Kagan et al., 2017). Pharmacological or genetic inhibition of either ACSL4 or LPCAT3 suppresses ferroptosis specifically over other forms of cell death (Dixon et al., 2015; Yuan et al., 2016b; Doll et al., 2017; Kagan et al., 2017). Lipoxygenases can catalyze the formation of lipid hydroperoxides from PUFAs, instigating toxic lipid peroxidation (Yang et al., 2016), although this can occur independently from lipoxygenase activity (Shah et al., 2017).

As mentioned above, the precise role of iron in ferroptosis remains unclear. The ability of the iron chelators including deferoxamine and deferiprone to salvage cells from ferroptotic death in a variety of models underscores the role of iron in triggering ferroptosis (Dixon et al., 2012; Friedmann Angeli et al., 2014; Yang et al., 2014; Do Van et al., 2016). The lipoxygenases involved in generating toxic lipid hydroperoxides require catalytic iron in their active sites (Abeysinghe et al., 1996), hence the protective effect of iron chelation has been proposed to involve inhibition of lipoxygenase activity by removal of the essential catalytic iron from these enzymes. Alternatively, iron has been demonstrated to potentiate ferroptosis in a free radical-mediated process independent from lipoxygenase activity (Shah et al., 2017).

Ferroptosis can be experimentally induced by direct inhibition of Gpx4 via Ras-selective lethal small molecule 3 (RSL3) or by genetic knockdown or deletion of Gpx4 (Friedmann Angeli et al., 2014; Yang et al., 2014). As Gpx4 requires glutathione as a substrate, ferroptosis is also induced by disruption of glutathione supply via inhibition of glutathione synthesis (e.g., buthionine sulfoximine) or inhibiting the supply of cysteine required for glutathione synthesis via inhibition of the cystine-glutamate antiporter system x_c_^-^ (via erastin, sulfasalazine, or sorafenib) (Dixon et al., 2012; Friedmann Angeli et al., 2014) (**Figure 1**).

Mitochondria play key roles in regulated cell death (Galluzzi et al., 2018), and ferroptosis is no exception. Cells undergoing ferroptosis exhibit specific mitochondrial morphology. Early studies using the system x_c_^-^ inhibitor erastin show ferroptosis results in smaller mitochondria with increased mitochondrial membrane density (Yagoda et al., 2007; Dixon et al., 2012). Later studies employing pharmacological or genetic disruption of Gpx4 causes mitochondrial swelling, decreased cristae and outer membrane rupture (Friedmann Angeli et al., 2014; Doll et al., 2017; Maiorino et al., 2017; Ingold et al., 2018). Toxicity induced by inhibition of mitochondrial complex I can be rescued by ferroptosis inhibitors (Basit et al., 2017). Furthermore, lack of ACSL4 prevents the RSL3-mediated rupture of mitochondrial outer membrane (Doll et al., 2017), while knockdown of mitochondrial acyl-CoA synthetase family member 2 (ACSF2; involved in fatty acid metabolism) prevents erastin toxicity, suggesting ACSF2 generates a mitochondrial-specific lipid necessary for ferroptosis (Dixon et al., 2012).

Despite these observations, evidence for mitochondrial lipid peroxidation during ferroptosis is mixed. Ferroptosis induced by erastin does not appear to be accompanied by mitochondrial lipid peroxidation *in vitro* (Dixon et al., 2012; Do Van et al., 2016) or *in vivo* (Wang et al., 2016). In contrast, disruption of Gpx4 results in mitochondrial lipid peroxidation *in vitro* and in kidneys (Friedmann Angeli et al., 2014). This discrepancy suggests that the inducing stimuli may be critical for the subcellular localization of lipid peroxidation.

Although mitochondria are clearly impaired in ferroptosis, evidence suggests that they are not driving the cell death process. Cells deficient in mitochondria remain sensitive to ferroptosis (Gaschler et al., 2018). Furthermore, extramitochondrial lipid peroxidation temporally precedes mitochondrial lipid peroxidation, and mitochondrial damage including rupture of the outer mitochondrial membrane is a late event, closely preceding cell lysis (Friedmann Angeli et al., 2014; Jelinek et al., 2018).

Reports on targeting antioxidants to mitochondria are mixed. MitoQ rescues neuronal cells from RSL3 toxicity (Jelinek et al., 2018). However, when compared their non-mitochondrial analogs, mitochondrially targeted radical scavengers are opposingly reported as being less effective (Friedmann Angeli et al., 2014) or more effective (Krainz et al., 2016).

Mitochondrial iron is also implicated in ferroptosis. MitoNEET, also known as CISD1, is an iron-containing outer mitochondrial membrane protein involved in iron export from mitochondria (Mittler et al., 2018). Knockdown of mitoNEET exacerbates erastin toxicity and increases mitochondrial iron content and lipid peroxidation, whereas stabilization of mitoNEET attenuates erastin toxicity and decreases mitochondrial lipid peroxidation (Yuan et al., 2016a). Alternatively, safely sequestering iron within mitochondria via overexpression of mitochondrial ferritin is able to curb erastin-induced cell death, both *in vitro* and *in vivo* (Wang et al., 2016).

## Evidence for Ferroptosis in Neurodegeneration

Explicitly identifying ferroptosis *in vivo* is hampered by the lack of specific biomarkers. Nevertheless, considerable evidence exists that implicates ferroptosis in neurodegeneration. The association between oxidative stress, lipid peroxidation and neurodegeneration has long been appreciated. Notably, elevated levels of lipid peroxidation are reliably detected in brain tissues and body fluids of Alzheimer’s, Parkinson’s, Huntington’s disease, motor neuron disease and multiple sclerosis patients (Adibhatla and Hatcher, 2010; Shichiri, 2014; Sugiyama and Sun, 2014; Wang et al., 2014; Bradley-Whitman and Lovell, 2015). Iron accumulation is a consistent feature of neurodegeneration (Belaidi and Bush, 2016). The level of iron in brains of individuals with mild cognitive impairment and Alzheimer’s disease correlates with disease progression (Smith et al., 2010; Ayton et al., 2017). Elevated iron is a cardinal feature of Parkinson’s disease substantia nigra (Ayton and Lei, 2014), and increased iron is detected in affected brain regions of patients with motor neuron disease, multiple sclerosis, Huntington’s disease and Friedreich ataxia (Kwan et al., 2012; Li and Reichmann, 2016; Sheykhansari et al., 2018). Reducing brain iron via the chelators deferiprone or deferoxamine is efficacious in clinical trials of Parkinson’s (Devos et al., 2014) and Alzheimer’s patients (Crapper McLachlan et al., 1991), respectively, indicating iron is contributing to the disease process. Further indirect evidence, including diminished glutathione and insufficient Nrf2 signaling (see below), is consistent with the presence of ferroptosis in neurodegeneration (Liddell, 2017; Liddell and White, 2017). Moreover, impaired mitochondrial function is common to many neurodegenerative diseases (Carri et al., 2017; Liddell and White, 2017; Liot et al., 2017; Swerdlow, 2017). Morphologically, mitochondria in brains of mice modeling Huntington’s disease exhibit disrupted cristae (Lee et al., 2011), while those in motor neuron disease human postmortem tissue and model mice feature swollen and vacuolated mitochondria (Jaarsma et al., 2000; Cozzolino and Carri, 2012) reminiscent of the mitochondrial changes evident in ferroptosis.

Since its original characterisation in cancer cells, the concept of ferroptosis has instigated growing efforts to explicitly detect and measure its footprint in neurodegeneration (Guiney et al., 2017; Morris et al., 2018). In this regard, more direct evidence for the role of ferroptosis in neurodegeneration has recently been generated. Neuronal cells are sensitive to erastin and RSL3 toxicity *in vitro*. This toxicity is associated with mitochondrial impairments, and is rescued by ferroptosis inhibitors or a mitochondrially targeted ROS scavenger (Neitemeier et al., 2017; Jelinek et al., 2018). Genetic models demonstrate the substantial reliance of neurons on Gpx4 to prevent toxic lipid peroxidation. Whereas global deletion of Gpx4 is embryonic lethal (Imai et al., 2003), mice with targeted mutation of Gpx4 selenocysteine to cysteine (sensitive to inactivation), or knockout of Gpx4 specifically in neurons are viable but exhibit selective loss of CA3 hippocampal interneurons, resulting in seizures and early death (Seiler et al., 2008; Ingold et al., 2018). Targeting Gpx4 knockout to photoreceptor neurons results in death of these cells within 21 days of birth (Ueta et al., 2012). Post-development, conditional knockout of Gpx4 in adult mice results in loss of CA1 hippocampal neurons and rapid death, indicating neurons are specifically sensitive to Gpx4 deletion (Yoo et al., 2012). Conditional ablation of Gpx4 targeted to neurons results in dramatic degeneration of motor neurons that rapidly progresses to paralysis and death (Chen et al., 2015). Targeting conditional knockout of Gpx4 to forebrain neurons of adult mice causes cognitive impairments and hippocampal degeneration reminiscent of Alzheimer’s disease (Hambright et al., 2017). These models are all accompanied by lipid peroxidation and mitochondrial impairments, consistent with ferroptosis. Conditional Gpx4 knockout can partially rescued by ferroptosis inhibitors, indicating the involvement of ferroptosis (Chen et al., 2015; Hambright et al., 2017).

Ferroptosis inhibitors have also been investigated in explicit models of neurodegneration. Ferroptosis inhibitors prevent the toxicity of Huntington’s disease-associated mutated Htt in brain slice cultures (Skouta et al., 2014). Ferroptosis inhibitors are also protective in dopaminergic cultured cells, organotypic slice cultures, and in an MPTP mouse model of Parkinson’s disease (Do Van et al., 2016). Furthermore, ferroptosis is implicated in hemorrhagic and ischemic stroke based on the ability of ferroptosis inhibitors to protect against neuronal death in both *in vivo* and *in vitro* models (Li et al., 2017; Tuo et al., 2017; Zille et al., 2017). Ischemic stroke is also strongly modulated by brain iron levels (Tuo et al., 2017). Knockout of glutathione synthesis accelerates the disease phenotype in motor neuron disease-model mice, and ultrastructural analysis of spinal cord tissue reveals mitochondrial swelling, rupture and decreased cristae, all consistent with ferroptosis (Vargas et al., 2011).

Ferroptosis also appears to mechanistically overlap with other cell death modalities in neurodegeneration. Consistent with a distinct form of cell death, it was initially reported that ferroptosis induced by erastin does not involve mitochondrial genes implicated in other cell death pathways, nor the release of cytochrome c (Dixon et al., 2012). However, recent studies show that ferroptosis induces the pro-apoptotic translocation of BH3 interacting-domain death agonist (BID) to mitochondria in neuronal cells (Neitemeier et al., 2017; Jelinek et al., 2018). Oxytosis is described as glutamate-induced inhibition of cystine uptake via system x_c_^-^ leading to glutathione depletion and subsequent lipoxygenase-dependent toxic lipid peroxidation (Tan et al., 2001). This is clearly very similar to ferroptosis and has led to the recent proposal that perhaps ferroptosis and oxytosis are in fact the same pathway (Lewerenz et al., 2018). Accordingly, oxytosis in neurons causes mitochondrial morphological changes similar to ferroptosis (Lee et al., 2011), and the toxicity of glutamate mirrors that of erastin or RSL3 in neuronal cells, and are all amenable to prevention by ferroptosis inhibitors (Liu et al., 2015; Neitemeier et al., 2017; Jelinek et al., 2018) or necrostatin-1 (Xu et al., 2007). Furthermore, oxytosis/ferroptosis in neurons involves the translocation of apoptosis inducing factor (AIF) from mitochondria to nucleus, prevention of which alleviates cell death (Xu et al., 2010; Neitemeier et al., 2017; Jelinek et al., 2018). To further complicate the mechanisms of cell death, mitochondrial AIF release is the penultimate stage of parthanatos, a poly ADP-ribose polymerase 1 (PARP-1)-mediated form of cell death (Fricker et al., 2018; Galluzzi et al., 2018). Parthanatos can be induced by oxidative stress, and is also implicated in neurodegeneration (Fricker et al., 2018; Galluzzi et al., 2018). Hence both initiating events and late stages are shared by ferroptosis/oxytosis and parthanatos.

Together these studies show that many features facilitating ferroptosis are present in neurodegeneration, and mounting evidence indicates that targeting ferroptosis with specific inhibitors is a valid therapeutic strategy. However, overlapping mechanisms highlight the need for more comprehensive delineation of the cellular mechanisms of cell death, and the potential for new or repurposed treatments. An alternate approach to attenuate ferroptosis is to augment the endogenous anti-ferroptotic mechanisms within cells. To this end, the role of the transcription factor Nrf2 will now be discussed.

## Is Nrf2 an Anti-Ferroptosis Transcription Factor?

In terms of endogenous cellular mechanisms preventing ferroptosis, the antioxidant transcription factor nuclear factor erythroid 2-related factor 2 (Nrf2) is exquisitely positioned to modulate the onset and outcomes of ferroptosis. Nrf2 is responsible for regulating hundreds of antioxidant genes (Gao et al., 2014). Under normal conditions, Nrf2 resides in the cytosol bound to its negative regulator Kelch-like ECH-associated protein 1 (Keap1). Keap1 constitutively targets Nrf2 for ubiquitination and proteasomal degradation, thereby maintaining Nrf2 signaling capacity at a low level. However, states of increased oxidative stress facilitate the dissociation of Nrf2 from Keap1, and promote the nuclear translocation of Nrf2. In the nucleus, Nrf2 interacts with antioxidant response elements (AREs) in the promoter region of target genes resulting in their transcriptional activation (Yamamoto et al., 2018).

Importantly in the context of ferroptosis, almost all genes thus far implicated in ferroptosis are transcriptionally regulated by Nrf2 (**Table 1**). These include genes for glutathione regulation (synthesis, cysteine supply via system x_c_^-^, glutathione reductase, glutathione peroxidase 4), NADPH regeneration which is critical for Gpx4 activity (glucose 6-phosphate dehydrogenase, phosphogluconate dehydrogenase, malic enzyme), and iron regulation (including iron export and storage, heme synthesis and catabolism) (Sasaki et al., 2002; Lee et al., 2003; Wu et al., 2011; Kerins and Ooi, 2017). In addition, Nrf2 is involved in the regulation of lipids via the ligand-mediated transcription factor peroxisome proliferator-activated receptor gamma (PPARγ). Nrf2 and PPARγ are reciprocally regulated, with activation of either upregulating the other (Cai et al., 2017; Lee, 2017). PPARγ is a major regulator of lipid metabolism (Cai et al., 2017), and can be activated by oxidized lipids relevant to the initiation of ferroptosis (Itoh et al., 2008). Hence Nrf2 indirectly modulates the lipids whose abundance contributes to the sensitivity to ferroptosis (Doll et al., 2017) (**Figure 1**).

**Table 1:** Ferroptosis-related genes that are transcriptionally regulated by Nrf2.

Gene symbol	Gene name	Reference
**Glutathione-related**		
GCLM	Glutamate-cysteine ligase modifier subunit	Thimmulappa et al., 2002; Kwak et al., 2003; Lee et al., 2003
GCLC	Glutamate-cysteine ligase catalytic subunit	Thimmulappa et al., 2002; Kwak et al., 2003; Lee et al., 2003; Wu et al., 2011
GSS	Glutathione synthetase	Thimmulappa et al., 2002; Wu et al., 2011
SLC7A11	System x_c_^-^	Sasaki et al., 2002
GPX4	Glutathione peroxidase 4	Lee et al., 2003; Salazar et al., 2006; Wu et al., 2011
GSR	Glutathione reductase	Thimmulappa et al., 2002; Kwak et al., 2003; Lee et al., 2003; Wu et al., 2011
TXN1	Thioredoxin 1	Kwak et al., 2003; Lee et al., 2003
TXNRD1	Thioredoxin reductase 1	Kwak et al., 2003; Lee et al., 2003; Wu et al., 2011
**Iron-related**		
FTH1	Ferritin heavy chain 1	Lee et al., 2003; Pietsch et al., 2003; Wu et al., 2011
FTL	Ferritin light chain	Thimmulappa et al., 2002; Lee et al., 2003; Pietsch et al., 2003; Wu et al., 2011
TFRC	Transferrin receptor 1	Lee et al., 2003
FPN1 (SLC40A1)	Ferroportin	Marro et al., 2010
MT1G	Metallothionein 1G	Sun et al., 2016a
HMOX1	Heme oxygenase 1	Alam et al., 1999; Lee et al., 2003
BLVRA	Biliverdin reductase A	Agyeman et al., 2012
BLVRB	Biliverdin reductase B	Wu et al., 2011; Agyeman et al., 2012
FECH	Ferrochelatase	Wu et al., 2011; Campbell et al., 2013
ABCB6	ATP binding cassette subfamily B member 6	Campbell et al., 2013
HRG1	Heme-response gene 1	Campbell et al., 2013
**NAPDH regeneration**		
G6PD	Glucose-6-phosphate dehydrogenase	Thimmulappa et al., 2002; Lee et al., 2003; Wu et al., 2011
PGD	Phosphogluconate dehydrogenase	Thimmulappa et al., 2002; Wu et al., 2011
ME1	Malic enzyme 1	Thimmulappa et al., 2002; Kwak et al., 2003; Lee et al., 2003; Wu et al., 2011
**Transcription factors**		
PPARG	Peroxisome proliferator-activated receptor gamma	Cho et al., 2010
PPARGC1A	Peroxisome proliferator-activated receptor gamma coactivator 1α	Neymotin et al., 2011

Nrf2 also plays important roles in modulating mitochondrial function. Nrf2 can be physically bound to mitochondria and thus monitor and respond to changes in mitochondrial function (Lo and Hannink, 2008). Nrf2 also regulates mitochondrial dynamics, including biogenesis (Piantadosi et al., 2008; Merry and Ristow, 2016) via interaction with peroxisome proliferator-activated receptor gamma coactivator 1α (PGC-1α) (Dinkova-Kostova and Abramov, 2015; Navarro et al., 2017), and mitophagy via a P62-dependent, PINK1/Parkin-independent mechanism (East et al., 2014). Mitochondria in Nrf2 knockout mice have impaired function, whereas activation of Nrf2 enhances mitochondrial function and resistance to stressors (Greco and Fiskum, 2010; Neymotin et al., 2011; Holmstrom et al., 2016).

Therefore, by virtue of its direct and indirect regulation of many genes central to ferroptosis and its regulation of mitochondrial function, Nrf2 is positioned to be a key player in ferroptosis. Despite this, the role of Nrf2 in ferroptosis has received very little attention thus far, limited mainly to *in vitro* studies using cancer cells. As expected, Nrf2 activation confers resistance to ferroptosis in cancer cells (Sun et al., 2016b; Chen et al., 2017a,b; Roh et al., 2017). Genetic modulation of Nrf2 expression, including knockdown and overexpression, can fine-tune the sensitivity of glioma cells to the ferroptosis inducers erastin and RSL3 (Fan et al., 2017). This may contribute to the cancer-promoting and chemoresistance effects of elevated Nrf2 present in many cancers (Fan et al., 2017; Taguchi and Yamamoto, 2017).

Nrf2 activation is sensitive to shifts in cellular redox status, hence it follows that the lipid peroxidation accompanying ferroptosis should activate Nrf2. Indeed, the ferroptosis inducers erastin and sorafenib are sufficient to activate Nrf2 in hepatocellular carcinoma cells (Houessinon et al., 2016; Sun et al., 2016b). Furthermore, the protective action of the mitochondrially targeted antioxidant, MitoQ, involves Nrf2 activation (Hu et al., 2018).

Given the pervasive lipid peroxidation evident in neurodegeneration, it would be expected that Nrf2 would be elevated in neurodegenerative diseases. While there is some evidence for Nrf2 activation in neurodegeneration, it appears to be relatively mild and clearly insufficient to prevent neuronal dysfunction (Liddell, 2017). In some cases, particularly for motor neuron disease, Nrf2 signaling appears to be impaired (Moujalled et al., 2017). This is in contrast to the strong activation of Nrf2 evident when genetic or pharmacological Nrf2 inducers are applied to models of neurodegeneration (Liddell, 2017). These treatments are robustly neuroprotective in animal models of disease. While several drugs targeting Nrf2 are currently under clinical investigation, to date, dimethyl fumarate (Tecfidera) remains the only clinically approved drug for the treatment of a neurodegenerative disease (relapsing-remitting multiple sclerosis) in which Nrf2 activation clearly contributes to its mechanism of action (Havrdova et al., 2017).

The above mentioned studies in cancer cells provide empirical *in vitro* support for the anti-ferroptotic action of Nrf2. However, direct *in vivo* evidence for an anti-ferroptotic effect of Nrf2 induction does not yet exist, and it is currently unknown whether and to what extent the demonstrated neuroprotective efficacy of Nrf2 activation in models of neurodegeneration involve attenuation of ferroptosis. Evaluation of ferroptosis *in vivo* is currently hindered by the lack of specific markers of ferroptosis. Studies examining the effect of Nrf2 activation in explicit *in vivo* models of ferroptosis (e.g., Gpx4 knockout) would provide some insight.

## Conclusion and Future Perspectives

Ferroptosis is an iron-dependent form of regulated cell death instigated by impaired glutathione metabolism, culminating in mitochondrial failure and toxic lipid peroxidation. Emerging evidence implicates ferroptosis in neurodegeneration, both in the molecular and biochemical signatures of neurodegeneration, and in terms of functional abrogation of neuron death via specific ferroptosis inhibitors. Elucidating how ferroptosis provokes neurodegeneration will expose new therapeutic opportunities to treat these diseases. To this end, targeting the antioxidant transcription factor Nrf2 is an attractive option. Nrf2 signaling is involved in regulating mitochondrial function and impacts almost all identified molecular aspects of ferroptosis. Treatments targeting Nrf2 have been demonstrated to exert anti-ferroptotic effects in the context of cancer cells, and are beneficial in many models of neurodegeneration. The protective mechanism of Nrf2 activation in these models may involve attenuating ferroptosis via upregulation of the endogenous anti-ferroptotic machinery, however, direct evidence for this is currently lacking. The myriad of Nrf2 actions described here suggest that targeting Nrf2 is an exciting therapeutic option to attenuate ferroptosis.

Several questions remain unanswered. The discovery of bonafide biomarkers of ferroptosis will be invaluable to unequivocally probe its involvement in neurodegeneration and facilitate the development of therapeutic treatments targeting ferroptosis. The continued evaluation of ferroptosis inhibitors and comparison to inhibitors of other cell death modalities in further models of neurodegeneration will help elucidate the key pathways involved. Finally, whether Nrf2 activation is directly alleviating ferroptotic stress.

Taken together, the arguments presented in this review elucidate a coherent network that links Nrf2 signaling to mitochondrial function and ferroptotic cell death, and proposes the targeting of Nrf2 as a rational line of therapy for ferroptotic neurodegeneration.

## Author Contributions

JL conceived the review. MA prepared the first draft. RL, KK, and TM reviewed literature and contributed to writing. JL prepared the final manuscript.

## Conflict of Interest Statement

The authors declare that the research was conducted in the absence of any commercial or financial relationships that could be construed as a potential conflict of interest.
